# An Updated Review of Fossil Pollen Evidence for the Study of the Origin, Evolution and Diversification of Caribbean Mangroves

**DOI:** 10.3390/plants12223852

**Published:** 2023-11-14

**Authors:** Valentí Rull

**Affiliations:** 1 Botanic Institute of Barcelona, Spanish National Research Council (CSIC), Pg. del Migdia s/n, 08028 Barcelona, Spain; vrull@csic.es; 2 Institut Català de Paleontologia Miquel Crusafont, Universitat Autònoma de Barcelona, C. de les Columnes s/n, 08193 Cerdanyola del Vallès, Spain

**Keywords:** Caribbean mangroves, origin, evolution, turnover, diversification, fossil pollen, Eocene, Oligocene, Miocene, Pliocene

## Abstract

Recently, the evolutionary history of the Caribbean mangroves has been reconsidered using partial palynological databases organized by the time intervals of interest, namely Late Cretaceous to Eocene for the origin, the Eocene–Oligocene transition for major turnover and Neogene to Quaternary for diversification. These discussions have been published in a set of sequential papers, but the raw information remains unknown. This paper reviews all the information available and provides the first comprehensive and updated compilation of the abovementioned partial databases. This compilation is called CARMA-F (CARibbean MAngroves-Fossil) and includes nearly 90 localities from the present and past Caribbean coasts, ranging from the Late Cretaceous to the Pliocene. Details on the Quaternary localities (CARMA-Q) will be published later. CARMA-F lists and illustrates the fossil pollen from past mangrove taxa and their extant representatives, and includes a map of the studied localities and a conventional spreadsheet with the raw data. The compilation is the most complete available for the study of the origin, evolution and diversification of Caribbean mangroves, and is open to modifications for adapting it to the particular interests of each researcher.

## 1. Introduction

**Figure 1:**
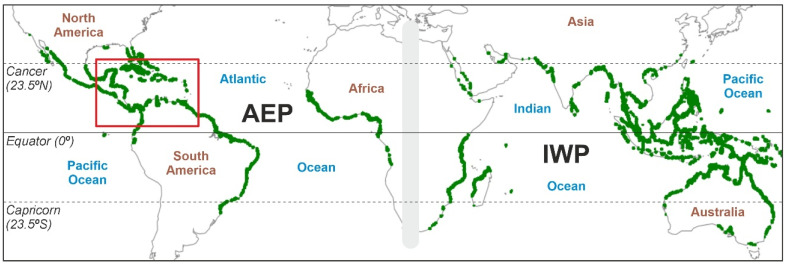
Worldwide distribution of mangroves (green fringes), with the Caribbean region highlighted by a red box. The barrier between the AEP and IWP biogeographical regions is represented as a gray band. AEP, Atlantic–East Pacific region; IWP, Indo-West Pacific region. Base map from Ref. [10].

Mangroves are intertidal ecosystems that develop a worldwide forested fringe along tropical/subtropical coasts between approximately 25° N and 25° S (Figure 1). Structurally, these ecosystems are organized around a number of tree species from varied orders and families that confer mangrove formations, their characteristic physiognomy, which has been considered an example of evolutionary convergence among taxonomically distant clades [1]. In addition to their intrinsic value as natural systems, mangroves are important for the following reasons: (i) they protect coasts and coastal ecosystems, such as corals, seagrasses and salt marshes, from erosion, thus favoring seaward progradation; (ii) they play a key role in the maintenance of biodiversity and ecological dynamics across the marine/terrestrial ecotone; (iii) they provide relevant ecological and cultural services (fisheries, cultivation, aquaculture, timber, fuel, aesthetics, ecotourism, etc.); and (iv) they are among the most efficient blue-carbon ecosystems that contribute to alleviating atmospheric CO_2_ increases by sequestering carbon in their organic-rich sediments [2,3,4,5,6,7,8,9].

However, mangroves are among the world’s most threatened ecosystems [11]. According to the latest estimates, the global mangrove extent was reduced by 3.4% in less than 25 years (1996–2020) due to natural and anthropogenic deforestation [12]. If these rates are maintained, these ecosystems will be severely reduced during this century, and their long-term survival is at great risk [13], which would imply increasing coastal erosion rates and biodiversity depletion, as well as losses in ecological and cultural services and in the global warming mitigation capacity. This has fostered the launching of numerous worldwide initiatives for mangrove conservation and restoration, which need sound ecological knowledge [14,15,16,17]. Most of these initiatives have been based on present-day ecological studies, but paleoecological research may also be useful, as it provides first-hand empirical evidence on the actual response of mangrove ecosystems to environmental (notably climatic, eustatic and anthropogenic) drivers of ecological change. This allows for the characterization of the main threats and helps define the corresponding response thresholds, thus providing information useful for mangrove conservation and management. Evolutionary studies are also valuable, as they furnish straightforward evidence on the evolutionary potential of mangrove species and their capacity to undergo genetic changes in response to environmental shifts [18].

**Figure 2:**
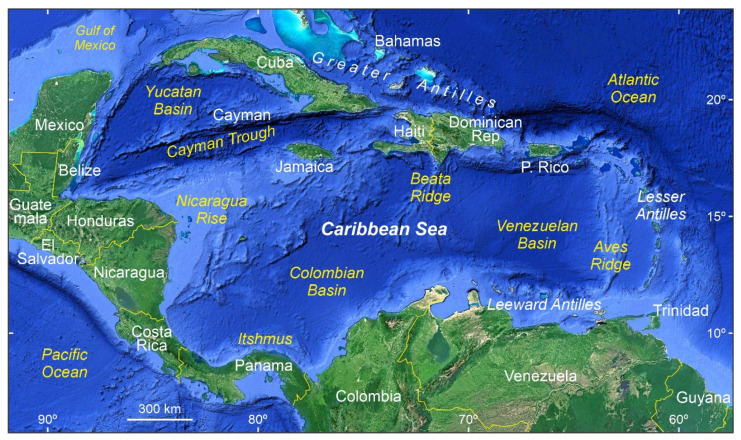
The Caribbean region, as considered in this paper, and its main physiographical features. Base map from Google Earth.

The Caribbean region (Figure 2) has been considered the cradle of Neotropical mangroves and a biodiversity hotspot for these ecosystems [19,20,21]. Current estimates for mangrove loss in the region are similar to global figures, and several conservation actions have been proposed specifically for the region [15]. In this context, the Caribbean mangroves were considered direct descendants of former pantropical Cretaceous mangroves that experienced regional differentiation after the closure of the Tethys Sea. However, a detailed quantitative analysis of the evidence strongly suggested that the first Caribbean mangroves did not appear until the Middle Eocene and were ecological and evolutionary innovations that emerged de novo, rather than as a consequence of the regional differentiation of former hypothetical Late Cretaceous pantropical mangroves [22].

**Figure 3:**
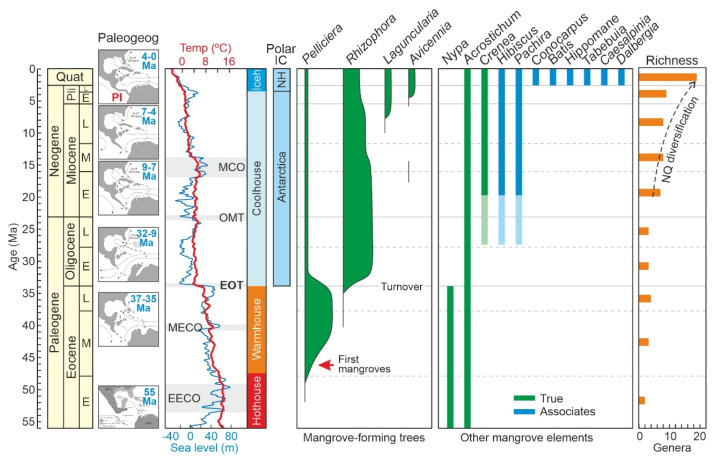
Summary of the main evolutionary trends of Caribbean mangroves, from their Eocene origin to their Neogene diversification, as analyzed and discussed in Refs. [1,18,22,23,24,25]. Paleogeographic reconstruction according to Ref. [26] and paleoclimatic/paleoesutatic curves according to Refs. [27,28]. Chronology: Quat, Quaternary; Pli, Pliocene; E, Early; M. Middle; L, Late. Paleogeography: PI, Panama Isthmus. Paleoclimates: EECO, Early Eocene Climatic Optimum; MECO, Middle Eocene Climatic Optimum; EOT, Eocene—Oligocene Transition; OMT, Oligocene/Miocene Transition; MCO, Miocene Climatic Optimum; Iceh, Icehouse; NQ, Neogene–Quaternary. Polar Ice Caps (IC): NH, Northern Hemisphere. Richness: NQ, Neogene–Quaternary.

The Eocene Caribbean mangroves were dominated by the ancestor of the extant *Pelliciera*, which was replaced by the ancestor of the modern *Rhizophora* after the Eocene–Oligocene transition (EOT), likely due to the global and intense cooling and sea-level fall that characterized this geological boundary [1]. The *Pelliciera* mangroves never returned, and their modern representatives remain as subordinate mangrove elements restricted to a small equatorial patch in Central America/NW South America [23]. The *Rhizophora* mangroves diversified during the Neogene and attained their present-like taxonomical composition in the Late Miocene–Pliocene after the emergence of *Avicennia* and *Laguncularia*, the other important mangrove-forming trees of extant Caribbean mangroves [24]. The Quaternary was a time of spatial and ecological reorganization due to the recurrent Pleistocene climatic/sea-level shifts, and the Holocene was characterized by the consequences of human disturbance, especially during the last 6000 years [25]. The last centuries have been characterized by a significant reduction in Caribbean mangrove cover due to natural and anthropogenic deforestation, which calls for urgent conservation/restoration actions [18]. A graphical summary of these events is provided in Figure 3.

These conclusions were based on partial datasets organized chronologically according to the time lapse of interest (i.e., Late Cretaceous to Eocene, EOT, and Neogene and Quaternary), which are available in the corresponding papers. A first attempt to synthesize all this information led to the development of a compilation called CARMA (CARibbean MAngroves), but only the main features of the existing fossil records were available, and the specific data remain unpublished [18]. The CARMA compilation has been further updated and subdivided into two conceptually different parts: a pre-Quaternary fossil section (CARMA-F) and a section containing Quaternary and modern records (CARMA-Q). This paper presents the most updated version of CARMA-F, which constitutes the basis for the study of Eocene origin, EOT evolutionary turnover and the Neogene diversification of Caribbean mangroves. The CARMA-Q update, useful for the study of modern mangroves in the face of Quaternary environmental shifts and their anthropization, is in progress and will be published later. In addition to providing a comprehensive view of the published information to unravel the origin and evolution of Caribbean mangroves, CARMA-F may be used as a guide for the interested researchers to locate the required data aimed at addressing their own particular interests. The present version of CARMA-F is fairly complete, considering the published data, and its content is consistent with the above evolutionary insights. However, the compilation remains open to new updates from future research, and further improvements, modifications and alternative hypotheses regarding the origin and evolution of Caribbean mangroves cannot be disregarded.

The paper is subdivided into three main sections. The first section briefly characterizes the extant Caribbean mangroves in terms of their taxonomic composition, whereas the second section illustrates the pollen of the main taxa, with emphasis on those with fossil representatives. The third section describes the CARMA-F compilation, which is provided as a spreadsheet in the Supplementary Material, the main geographical and chronological features of the localities studied, and the types of data provided in the original references, with illustrative examples of all of them. 

## 2. Extant Caribbean Mangroves

**Figure 4:**
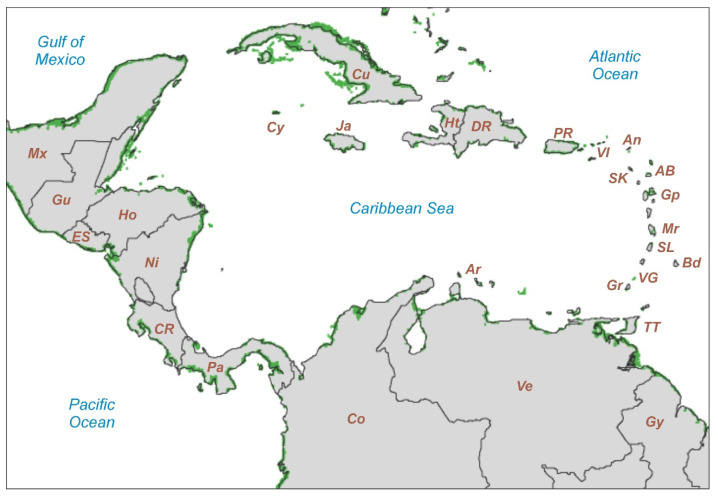
NASA Landsat 5-TM image of the Caribbean mangrove areas (green patches) using the data of Ref. [12]. Country/island abbreviations as in Table 1. Base map downloaded from https://earthobservatory.nasa.gov/images/47427/mapping-mangroves-by-satellite (accessed on 8 August 2023).

According to the latest estimates using remote sensing methods [12], Caribbean mangroves occupy a total extent of approximately 14,700 km^2^, which represents ~10% of the world’s total (Figure 4; Table 1). The countries with more extensive mangrove cover are Cuba, Venezuela, Colombia and Panama (1500–3600 km^2^); all other countries are below 750 km^2^, and 15 of them have less than 100 km^2^ of mangroves, with 9 below 10 km^2^ (Table 1).

Floristically, there are two main types of mangrove constituents: true (or strict) mangrove elements and mangrove associates (Table 2). The conditions for being considered a true mangrove element are the following [29]: (i) present only in mangroves, not extending into terrestrial communities; (ii) playing a major role in the structure of the community and able to form pure stands; (iii) having specific morphological adaptations to intertidal environments, typically pneumatophores and viviparous embryos; (iv) bearing physiological mechanisms for salt exclusion, as an adaptation to grow in saline waters; and (v) being systematically isolated from their terrestrial relatives, usually at the generic level, but often at the family/subfamily level.

**Figure 5:**
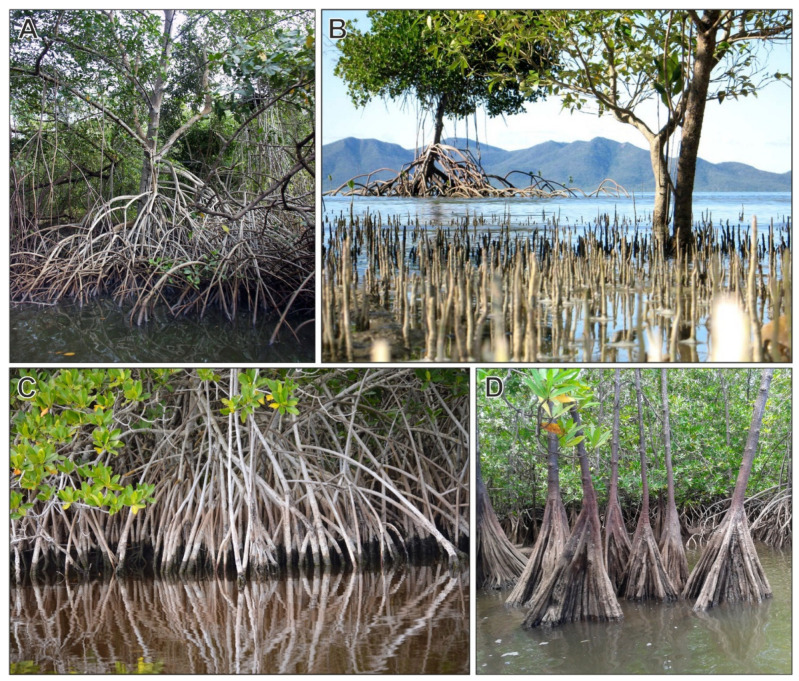
The main mangrove-forming tree species from the Caribbean region: (**A**) *Rhizophora mangle* (red mangrove); (**B**) *Avicennia germinans* (black mangrove); (**C**) *Laguncularia racemosa* (white mangrove); and (**D**) *Pelliciera rhizophorae* (tea mangrove). Modified from Ref. [25].

True mangrove elements are further subdivided into major and minor elements. Major true mangrove elements are mostly trees that are also known as mangrove-forming trees. In the Caribbean, the major true mangrove elements are of the genera *Rhizophora* (Rhizophoraceae), *Avicennia* (Acanthaceae) and *Laguncularia* (Combretaceae) (Figure 5). Minor true mangrove elements have similar traits but are not structurally important for the community (condition ii) and are unable to develop pure stands (iii), usually living in peripheral intertidal habitats. This is the case for *Pelliciera* (Tetrameristaceae) and *Acrostichum* (Pteridaceae) species, although the first can locally develop small pure stands under perhumid and shading conditions [31]). Mangrove associates are typical of mangrove environments but are not restricted to them (i), are not structurally important (ii) and lack morphological and physiological adaptations to intertidal habitats (iii, iv). These elements also occur in other habitats, such as coastal swamps, back-mangrove wetlands, salt marshes, riverbanks, beach communities and inland rainforests [29]. The herb *Crenea maritima* (Lythraceae) is exclusive to mangrove environments (i) and might be treated as a true mangrove element but it fails to meet conditions (ii) and (iii) and is therefore considered a mangrove associate. *Conocarpus erectus* is able to develop pure stands (ii) and is sometimes considered a true mangrove element, but it lacks morphological adaptations (iii) and does not tolerate flooding and high salinities (iv), thus living in marginal mangrove environments [32]. Some reviews on taxonomic, biogeographical, environmental and ecological features of some of the most important Caribbean true-mangrove elements have recently been published [32,33,34,35].

In addition to the above true and associate mangrove species, ~120 other accompanying species have been identified in the Neotropical mangroves, defining 30 phytosociological associations, all of which are present in the Caribbean region [36].

## 3. Modern and Fossil Pollen Types

**Figure 6:**
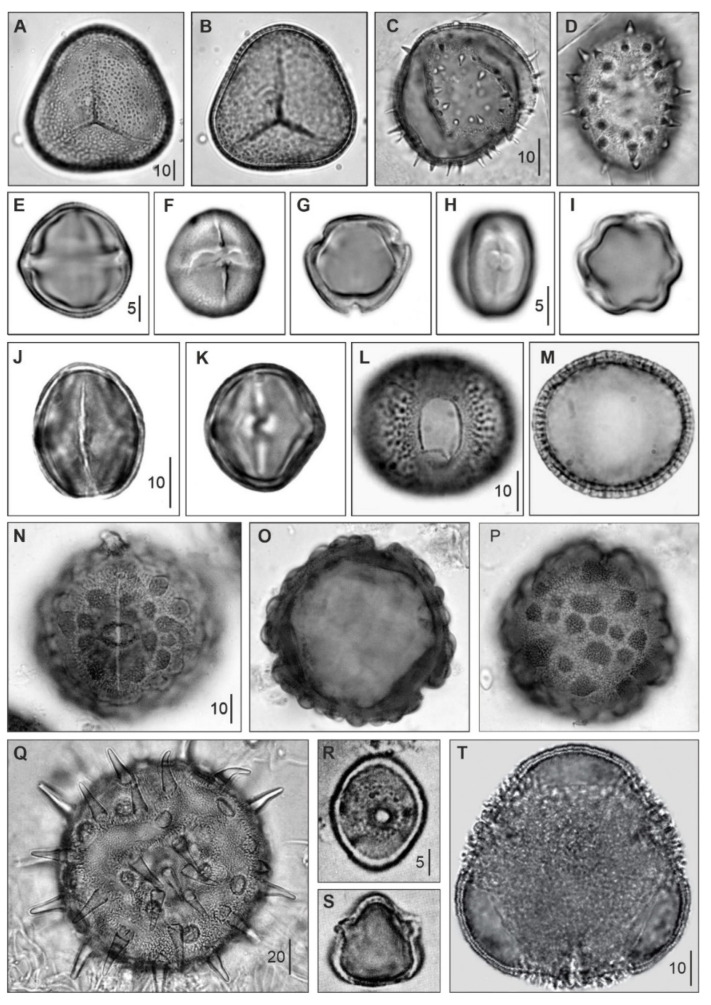
Pollen/spores from the main extant Caribbean mangrove species with fossil representatives (Table 3). (**A**,**B**), *Acrostichum aureum*; (**C**,**D**), *Nypa fruticans*; (**E**–**G**), *Rhizophora mangle*; (**H**,**I**), *Conocarpus erectus*; (**J**,**K**), *Laguncularia racemosa*; (**L**,**M**), *Avicennia germinans*; (**N**–**P**), *Pelliciera rhizophorae*; (**Q**), *Hibiscus tiliaceous*; (**R**,**S**), *Crenea patentinervis*; (**T**), *Pachira aquatica*. The palm *Nypa*, now restricted to the IWP region (Figure 1), is included because it was part of Caribbean mangroves until the Eocene [44,45]. Vertical bars are measurement scales in μm.

Fossil pollen/spores are, by large, the main evidence utilized in the evolutionary study of Caribbean mangroves. The pollen morphology of the main Caribbean mangrove components is illustrated in Figure 6, which is based on material from living plants and sedimentary pollen from modern sediments. It should be stressed that pollen morphology is rather homogeneous within each genus, and identification at the species level is not possible in most genera, with a few exceptions. This is why when dealing with pollen, we will refer to genera, except when some degree of morphological differentiation at the species level is possible. The generic names of extant mangrove components are usually extended to the whole Quaternary, assuming that they have been present during the last 2.6 Ma, which is a common procedure in Quaternary paleoecology [37]. In older sediments, where the occurrence of extant taxa is not guaranteed, artificial (as opposed to natural or living) species have been defined based on pollen morphology (morphospecies) and associated with extant genera, also on the basis of morphological identity. Since pollen morphology is a highly conservative character, from an evolutionary point of view [38,39], it has traditionally been assumed that these morphospecies represent the ancestors (likely at the generic level) of extant species, having similar ecological requirements. Indeed, paleoecological studies using fossils commonly rely on a reasonable degree of niche constancy over time (niche conservatism), especially at the genus level, in long-lasting communities [40,41,42,43], which is the case for mangroves.

This procedure, which has long been used in plant evolution, in general, and the Neotropics, in particular [46,47,48], has been validated by recent molecular phylogenetic studies, demonstrating that the main extant Caribbean mangrove genera were already present in the Paleogene, and that their modern species emerged mostly in the Neogene [19,49,50]. The fossil representatives of the main extant mangrove genera are listed in Table 3; the remaining true and associate mangrove genera (Table 2) do not have known Cretaceous, Paleogene or Neogene fossil equivalents and occur only in Quaternary and modern sediments. The palm *Nypa fruticans* Wurmb, now restricted to the IWP region, is included because it was present in the Caribbean region until the Eocene [44,45]. In this review, the names of extant genera are used as representatives of the corresponding lineages, according to the fossil representatives listed in Table 3.

**Table 3:** Paleogene and Neogene fossil pollen representatives of extant mangrove genera from the Caribbean region. Based on Refs. [45,51,52,53,54,55,56,57,58].

Genus	Fossil Representative (Morphospecies)	Range
Acacia *	*Polyadopollenites mariae* Dueñas	Paleogene–Neogene
*Acrostichum*	*Deltoidospora adriennis* (Potonié & Gelletich) Frederiksen	Cretaceous–Neogene
*Avicennia*	*Avicennia* *Retitricolporites* sp. Lorente	Neogene
*Crenea*	*Verrutricoporites rotundiporus* Van der Hammen & Wijsmtra	Neogene
*Hibiscus*	*Echiperiporites estelae* Germeraad, Hopping & Muller	Neogene
*Laguncularia*	*Laguncularia*	Neogene
*Nypa*	*Spinizocolpites echinatus* Muller, *S. baculatus* Muller *S. prominatus* (McIntyre) Stover & Evans	Cretaceous–Paleogene
*Pachira*	*Bombacacidites baculatus* Muller, Di Giacomo & Van Erve	Neogene
*Pelliciera*	*Psilatricolporites crassus* Van der Hammen & Wijsmtra *Lanagiopollis crassa* (Van der Hammen & Wijmstra) Frederiksen	Paleogene–Neogene
*Rhizophora*	*Zonocostites ramonae* Germeraad, Hopping & Muller *Zonocostites* spp.	Paleogene–Neogene

* Not included in Table 2 but considered to be a past mangrove associate by some authors [45].

## 4. The CARMA-F Compilation

**Figure 7:**
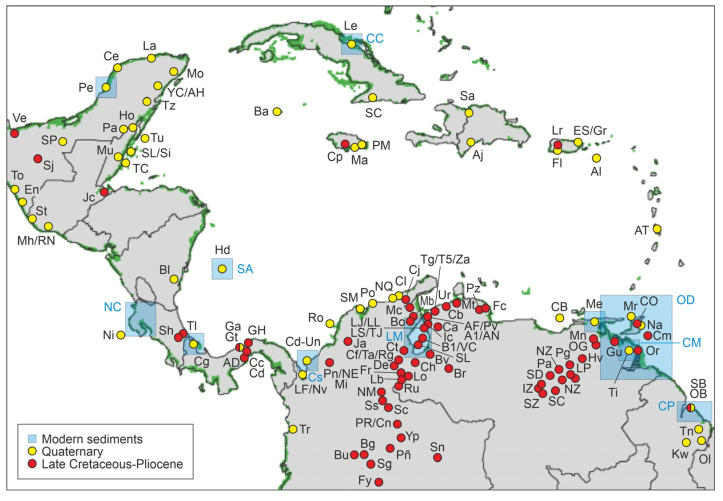
Localities with pollen records included in the CARMA compilation. Green areas represent the present extent of Caribbean mangroves [10]. Red dots mark the sites included in the CARMA-F section reviewed in this paper. Yellow dots (Quaternary records) and blue boxes (modern sediments) correspond to the CARMA-Q section, whose update is in progress. See the Supplementary Materials for locality names and original references.

The most updated CARMA version contains almost 160 entries/localities, of which 86 correspond to CARMA-F (Figure 7). The details on these localities and their fossil pollen data are provided in the Supplementary Material and are summarized as follows. Geographically, most fossil pollen sites (86%) are in the southern Caribbean coasts, especially in Colombia and Venezuela. This is due to the intensive and extensive exploration/production activities developed in these countries by the oil industry since the early 20th century. In these activities, fossil pollen played a key biostratigraphic role, especially in coastal and shallow-marine environments [51,57,59,60,61]. Many of the northern South American sites are located far from the present Caribbean coasts, but they were on near-mangrove coastal/shelf environments in the Paleogene and the Neogene. This is due to the highly dynamic paleogeography of the region driven mainly by the migration of the Caribbean plate and the occurrence of extensive marine incursions in NW South America [62,63,64,65]. The remaining CARMA-F localities lie in Central America (12%) and the Greater Antilles (2%), while the Lesser Antilles are devoid of fossil pollen records involving mangrove elements. The location of fossil records is approximate in many cases, especially in wells, due to the lack of coordinates, mostly for industrial confidentiality reasons. In these cases, the location of the records in Figure 7 has been placed according to maps and descriptions with the aid of Google Earth.

**Figure 8:**
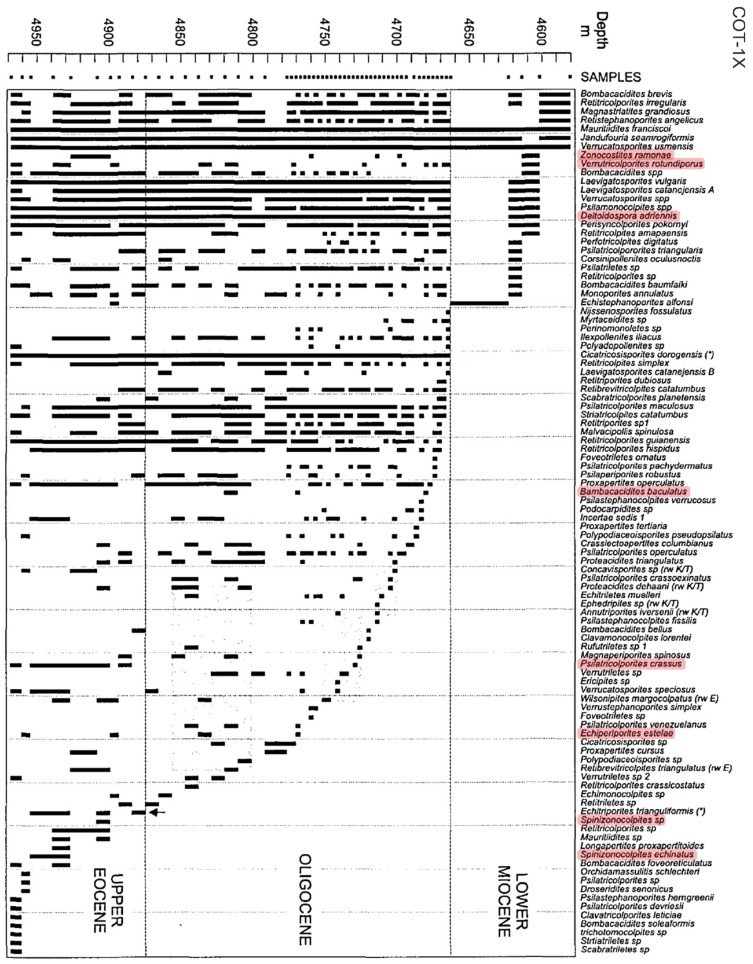
Range chart indicating the present/absence patterns in the Late Eocene–Early Miocene interval of well COT-1X from Venezuela (see Figure 7 for location and the Supplementary Materials for details). Mangrove representatives included in CARMA-F are highlighted in pink (see Table 3 for equivalences with extant taxa). Modified from Ref. [66].

**Figure 9:**
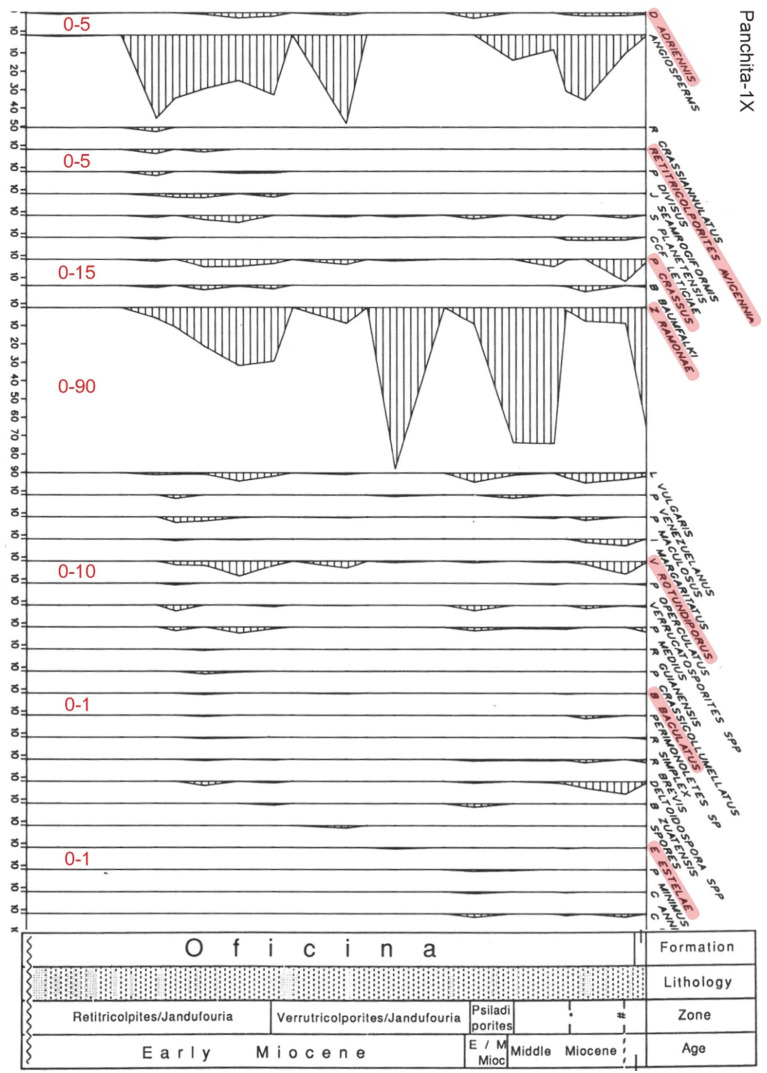
Percentage diagram of the Early Middle Miocene section of well Panchita-1X from Venezuela (Figure 7 and Supplementary Material), indicating the mangrove fossil pollen species highlighted in pink (Table 3). Values at the base of the diagram (in red) are the approximate percentage ranges used in the dataset. Modified from Ref. [57].

**Figure 10:**
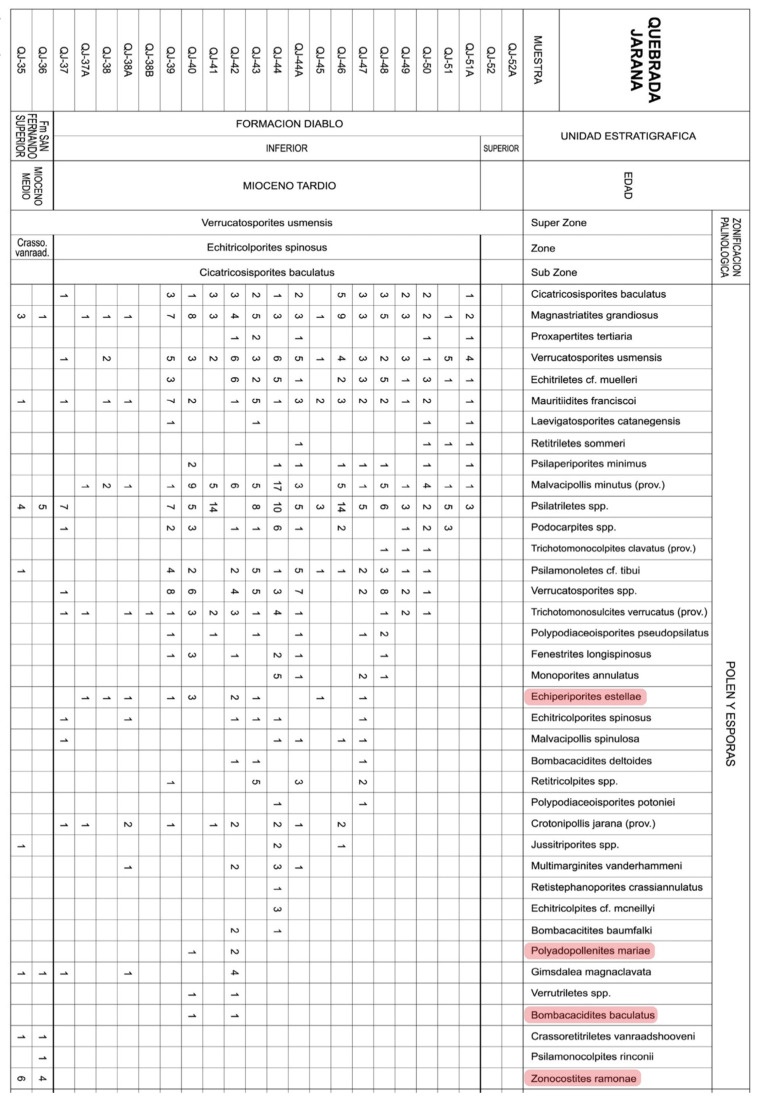
Raw counts of mangrove fossil pollen taxa (highlighted in pink) for the Middle–Late Miocene interval of the Quebrada Jarana in the Yopal site (Colombia) (Figure 7; Supplementary Material). Modified from Ref. [67].

**Figure 11:**
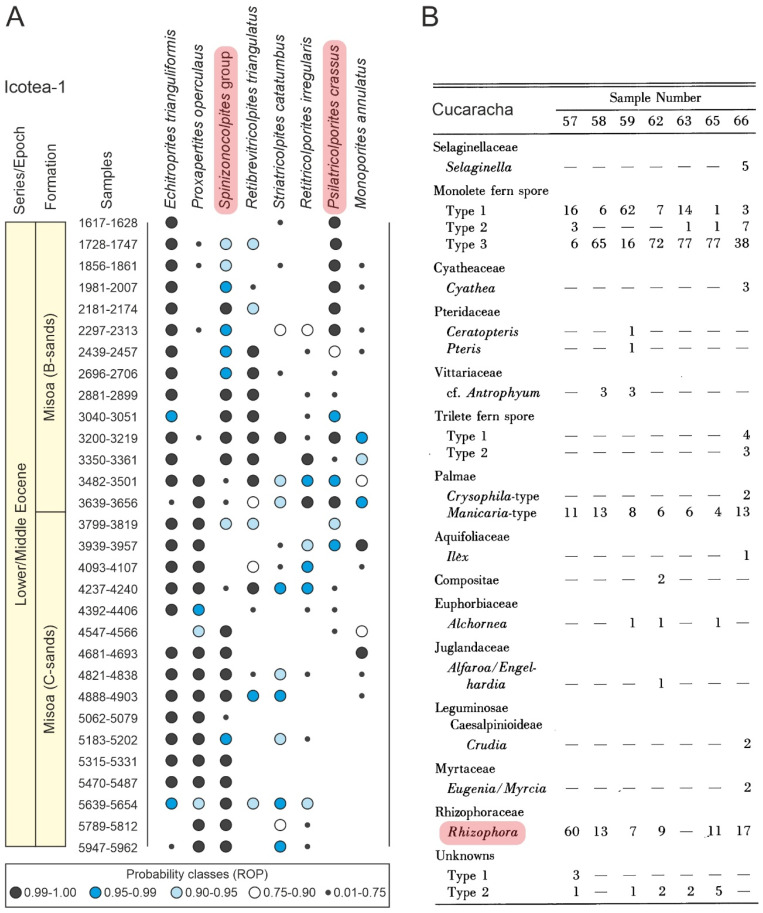
(**A**) Semiquantitative range chart of the Middle Eocene section of well Icotea-1 (Venezuela) using the re-observation probability (ROP). Modified from Ref. [51]. (**B**) Percentage table of the Early Miocene Cucaracha Formation (Panama). Modified from Ref. [68]. Mangrove taxa are highlighted in pink (see Figure 7, Table 3 and the Supplementary Material for location, botanical affinities and more details).

Chronologically, 6 localities bear Late Cretaceous sediments, 37 include Paleogene rocks, and 59 contain Neogene formations (this makes more than 86 items—actually 102—because a number of sections include combinations of these ages). The majority of records (61; 71%) provide quantitative data, usually pollen percentages but also raw counts in a few cases (5), whereas 19 (22%) report only presence, and 6 (7%) yield a semiquantitative parameter called re-observation probability (ROP), using the formula ROP = 1 − (1 − (a/N))^M^, where a = number of grains of a species counted in a sample, N = total number of grains of all species in the same sample, and M = total number of grains of all species in a new sample [51]. These data are displayed in several formats in the original references, namely, in-text taxa lists, tables and range charts for qualitative (presence/absence) data and diagrams or tables for percentages. ROP values are provided as range charts using symbols for probability classes. Illustrative examples of range charts, percentage tables/diagrams and ROP charts are provided in Figure 8, Figure 9, Figure 10 and Figure 11.

## 5. Final Remarks

The CARMA-F version presented here replaces the unpublished partial compilations used in previous papers [1,22,23,24,25], but the main conclusions in relation to the origin, evolution and diversification of Caribbean mangroves, as summarized in Rull [18] and synthesized in Figure 3, do not change. The refinements introduced by the updated dataset are addressed in detail in a book that will be issued next year [69]. The available version of CARMA-F is open to further additions and improvements and constitutes the most complete available compilation for studying any aspect of the origin and evolution of Caribbean mangroves. The format chosen for making the compilation public is a conventional spreadsheet so that interested researchers can freely use and modify this information according to their particular interests. As a former industry-based biostratigrapher, the author is aware that many palynological datasets potentially useful for the study of mangrove evolution remain unknown in confidential databases from oil companies. Some classical and highly cited papers, such as those by Germeraad et al. [51] or Lorente [57], among others, have demonstrated that it is possible to bring these data to light maintaining reasonable confidentiality rules. Continued efforts in this sense for the benefit of evolutionary knowledge would be acknowledged. Further improvements of CARMA-F would include the expansion of the compilation to the Caribbean/Gulf of Mexico region and eventually to the entire Neotropical region.

## Figures and Tables

**Table 1:** Mangrove cover by country/island in the Caribbean region. Raw data from Ref. [12], rounded to integers.

Country/Island	Map	Mangroves (km^2^)
Cuba	Cu	3597
Venezuela	Ve	2847
Colombia	Co	2808
Panama	Pa	1536
Nicaragua	Ni	747
Honduras	Ho	606
Belize	Bz	529
El Salvador	ES	373
Costa Rica	CR	371
Guyana	Gy	289
Guatemala	Gu	250
Dominican Republic	DR	192
Haiti	Ht	154
Jamaica	Ja	99
Puerto Rico	PR	83
Trinidad and Tobago	TT	82
Cayman Islands (UK)	Cy	45
Guadeloupe (France)	Gp	34
Martinique (France)	Mr	19
Antigua and Barbuda	AB	9
Virgin Islands (UK/USA)	VI	4
Grenada	Gr	2
Saint Lucia	SL	2
Anguilla (UK)	An	<1
Aruba	Ar	<1
Barbados	Bd	<1
Saint Kitts and Nevis	SK	<1
Saint Vincent and The Grenadines	VG	<1
**Total**		**14,677**

**Table 2:** True (major and minor) and associate mangrove plant elements of the Caribbean region. Based on Refs. [19,29,30]. Nomenclature according to the International Plant Names Index (IPNI) (https://www.ipni.org/ (accessed on 12 July 2023)).

Type		Species	Family	Plant Type
True	Major	*Avicennia bicolor* Standl. *	Acanthaceae	Tree
		*Avicennia germinans* (L.) Stearn *	Acanthaceae	Tree
		*Avicennia schaueriana* Stapf & Leechm. ex Moldenke *	Acanthaceae	Tree
		*Laguncularia racemosa* C.F.Gaertn. *	Combretaceae	Tree
		*Rhizophora mangle* L. *	Rhizophoraceae	Tree
		*Rhizophora racemosa* (G.Mey.) Engl. *	Rhizophoraceae	Tree
	Minor	*Acrostichum aureum* L.	Pteridaceae	Fern
		*Acrostichum daneaeifolium* Langsd. & Fisch. *	Pteridaceae	Fern
		*Pelliciera benthamii* (Planch. & Triana) N.C.Duke	Tetrameristaceae	Tree
		*Pelliciera rhizophorae* Planch. & Triana *	Tetrameristaceae	Tree
Associate		*Amphitecna latifolia* (Mill.) A.H.Gentry	Bignoniaceae	Tree
		*Anemopaegma chrysoleucum* (Kunth) Sandwith	Bignoniaceae	Vine
		*Batis maritima* L.	Batidaceae	Shrub
		*Caesalpinia bonduc* (L.) Roxb.	Fabaceae	Tree
		*Conocarpus erectus* L. *	Combretaceae	Tree
		*Crenea patentinervis* (Koehne) Standl. *	Lythraceae	Herb
		*Dalbergia ecastaphyllum* Taub.	Fabaceae	Tree/Shrub
		*Dalbergia amerimnum* Benth.	Fabaceae	Tree/Shrub
		*Hibiscus tiliaceus* L.	Malvaceae	Tree
		*Hippomane mancinella* L.	Euphorbiaceae	Tree
		*Mora oleifera* Duke *	Fabaceae	Tree
		*Muellera moniliformis* L.f. *	Fabaceae	Tree
		*Pachira aquatica* Aubl.	Bombacaceae	Tree
		*Pavonia rhizophorae* Killip *	Malvaceae	Shrub
		*Pavonia spicata* Cav.	Malvaceae	Shrub
		*Phryganocydia phellosperma* (Hemsl.) Sandwith	Bignoniaceae	Vine
		*Pluchea odorata* (L.) Cass.	Asteraceae	Herb
		*Rhabdadenia biflora* Müll.Arg.	Apocynaceae	Vine
		*Rustia occidentalis* (Benth.) Hemsl.	Rubiaceae	Tree/Shrub
		*Scaevola plumieri* (L.) Vahl	Goodeniaceae	Shrub
		*Tabebuia palustris* Hemsl. *	Bignoniaceae	Tree
		*Thespesia populnea* (L.) Sol. ex Corrêa	Malvaceae	Tree
		*Thespesia populneoides* (Roxb.) Kostel.	Malvaceae	Tree
		*Tuberostylis axilaris* S.F.Blake	Asteraceae	Shrub
		*Tuberostylis rhizophorae* Steetz	Asteraceae	Epiphyte

* Species used by Duke [19] to characterize the Atlantic–East Pacific (AEP) mangroves.

## Data Availability

Data are provided as Supplementary Material. The data are also publicly available at Mendeley Data (https://data.mendeley.com/datasets/zx8zvk3pw2/2; accessed on 12 November 2023).

## Supplementary Materials

The following supporting information can be downloaded at: https://www.mdpi.com/article/10.3390/plants12223852/s1. Refs. [70,71,72,73,74,75,76,77,78,79,80,81,82,83,84,85,86,87,88,89,90,91,92,93,94,95,96,97,98,99,100,101,102,103,104,105,106,107,108,109,110] are cited in the Supplementary Materials.
